# Herbal medicines in the treatment of children and adolescents with attention-deficit/hyperactivity disorder (ADHD): An updated systematic review of clinical trials

**DOI:** 10.22038/AJP.2022.21115

**Published:** 2023

**Authors:** Haide Golsorkhi, Mostafa Qorbani, Saeideh Sabbaghzadegan, Majid Dadmehr

**Affiliations:** 1 *Department of Traditional Medicine, School of Persian Medicine, Iran University of Medical Sciences, Tehran, Iran*; 2 *Student Research Committee, Iran University of Medical Sciences, Tehran, Iran*; 3 *Non-Communicable Diseases Research Center, Alborz University of Medical Sciences, Karaj, Iran*; 4 *Chronic Diseases Research Center, Endocrinology and Metabolism Population Sciences Institute, Tehran University of Medical Sciences, Tehran, Iran*; 5 *Institute for Studies in Medical History, Persian and Complementary Medicine, Iran University of Medical Sciences, Tehran, Iran*

**Keywords:** ADHD, Attention-deficit/hyperactivity disorder, Pediatric Herbal medicine, Systematic review

## Abstract

**Objective:**

This study was performed to provide an updated systematic review of herbal medicines and phytochemicals used for treatment of the pediatric patients with attention- deficit/hyperactivity disorder (ADHD).

**Materials and Methods:**

International electronic databases, including Scopus, PubMed, ScienceDirect, and Google Scholar were investigated from 1st January 2000 to late October 2021. Interventional studies published in English language, including randomized controlled trials (RCTs) or open-label clinical studies, which evaluated the effect of herbal medicines and phytochemicals on pediatric ADHD were included in this review.

**Results:**

Fifteen studies met the inclusion criteria. Several pieces of evidence support the efficacy of *Ginkgo biloba *L. and Pycnogenol; mainly inconclusive evidence could be found for *Valeriana officinalis *L*., Melissa officinalis *L., and ginseng. The results showed that while *Hypericum perforatum *L. was ineffective for ADHD, *Passiflora incarnata *L*., Crocus sativus *L, and *Prunus dulcis *(Mill.) D.A.Webb had similar efficacy compared to methylphenidate (MPH).

**Conclusion:**

A number of herbal medicines appear to be relatively safe and provide potential efficacy in amelioration of ADHD. However, due to lack of adequate reports of RCTs, no definitely specific recommendations could been made so far.

## Introduction

Attention-deficit/hyperactivity disorder (ADHD) is one of the most common neurodevelopmental disorders among the children and adolescents. According to the Diagnostic and Statistical Manual of Mental Disorders, Fifth Edition (DSM-5), ADHD is characterized by inattention, impulsivity, and hyperactivity (Pallucchini et al., 2021). This psychiatric disorder begins in childhood and is an important issue in mental health (Carol Ho et al., 2011). About 5-7% of school-aged children and 2-5% of adults are estimated to suffer from ADHD worldwide (Simon et al., 2009; Raman et al., 2018). Current treatment options principally include a pharmaceutical component, a behavioral component, and a psychosocial component separately or in combination (Sarris et al., 2011). However, some of children and adolescents with ADHD could not tolerate the side effects of psychostimulant medications (Brue and Oakland, 2002). Furthermore, concerns about risks of long- term exposure to these medications, such as dependence, diversion, and abuse discourage parents to continue the use of them (Heal et al., 2009). In recent years, the use of medicinal herbs has been considered an adjuvant to conventional pharmaceuticals in the management and amelioration of ADHD symptoms (Bussing et al., 2002). In order to evaluate the effectiveness and tolerability of herbal medicines for the management of ADHD, three systematic reviews have been published so far by Sarris et al. (2011), Wong et al. (2012), and Anheyer et al. (2017) (Sarris et al., 2011; Wong et al., 2012; Anheyer et al., 2017). Sarris et al. (2011) focused mainly on studies on the effect of nutritional interventions on ADHD rather than herbal interventions. Wong et al. (2012) have also only reviewed traditional oriental herbal medicines studies such as the Korean, Chinese and Japanese Pharmacopoeia on ADHD. Anheyer et al. (2017) reviewed evidence in herbal therapy in the treatment of pediatric ADHD, which were published up to July 2016. In the current update, we used more new studies based on a broader search to combine evidence in this scope to provide a basis for discussing the findings, and to identify areas that need further study. In addition, the results of studies on the efficacy of saffron, ginseng and sweet almond in the treatment of pediatric ADHD have been reviewed for the first time. Due to the importance of effective treatment with fewer side effects, this study was performed to evaluate current evidence related to safety and efficacy of the herbal medicines and phytochemicals on ADHD in an updated systematic review.

## Materials and Methods

The current systematic review was performed according to the standard of Preferred Reporting Items for Systematic Reviews and Meta-Analyzes (PRISMA) (Moher et al., 2015).

Search strategy

This systematic review of randomized clinical trials (RCTs) and open-label clinical studies was accomplished to determine the effect of herbal medicines and phytochemicals on the management and amelioration of ADHD in children and adolescents. Studies published in English language were investigated in electronic databases, including Scopus, PubMed, ScienceDirect and Google Scholar. The search was done using the following keywords: "attention deficit disorder with hyperactivity" [Title/Abstract] OR "ADHD" [Title/Abstract] OR impulsivity [Title/Abstract] AND child [Title/Abstract] OR children [Title/Abstract] OR adolescent [Title/Abstract] OR pediatric [Title/Abstract] OR paediatric [Title/Abstract] AND "Herbal Medicine" [Title/Abstract] OR "Plant Extracts" [Title/Abstract] OR "Herbal therapy" [Title/Abstract] OR "Safety" [Title/Abstract] OR "Efficacy" [Title/Abstract]. The search was performed independently by two researchers and the dispute was resolved by a third person. Reference lists of related review articles also were assessed for potential relevant studies.

Study selection and data extraction

All clinical trials or open-label (pilot) studies with a minimum of a 3-week duration that were published from 1st January 2000 to late October 2021, which assessed the effect of herbal medicines and phytochemicals on pediatric ADHD were included in the current review. The articles that studied ADHD participants under 18 years old were included (without any limitations on the diagnosis of ADHD). Combined type of ADHD as well as predominantly inattentive, and predominantly hyperactive/impulsive were considered ADHD. Therefore, all the participants in these clinical trials were pure ADHD. Other comorbidities, especially seizure/epilepsy were not included. Only studies that evaluated ADHD symptoms as a primary or secondary outcome were considered eligible. The cohort, case- control, case reports, duplicates, and unrelated studies as well as studies that did not provide required data were excluded. Chinese herbal medicines were not included in this study. Two review authors independently performed the screening of studies, and extracted the first author's name, study design, date of publication, sample size, age of participants, gender, intervention/comparison drug, outcomes, and data on the results and conclusion.

Risk of bias analysis

The Cochrane Collaboration Risk of bias tools (Higgins et al., 2011), which asses the sequence generation, the concealment of the allocation, blinding, and other biases were used for risk of bias analysis. The overall quality of each study was considered low risk of bias, some concerns of bias, or high-risk of bias. Therefore, if a domain with a high risk of bias is rated, the study is considered to have a high risk of bias. Disagreements between reviewers were discussed until consensus was reached. The risk of selection bias, reporting bias, attrition bias, performance bias and detection bias was assessed by the mentioned tool. According to the results of risk of bias analysis, the manuscripts were divided into three subgroups that include low, unclear, or high risk of bias.

## Results

Study characteristics and risk of bias among the included studies

**Figure 1:**
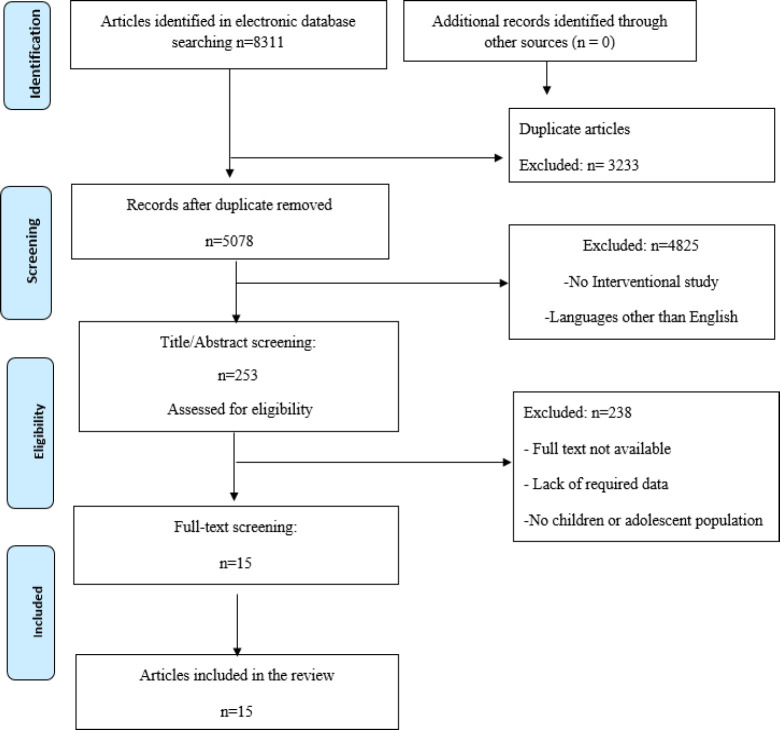
PRISMA (Preferred Reporting Items for Systematic Reviews and Meta-Analyses) flow diagram for the studies included in the current systematic review

**Figure 2:**
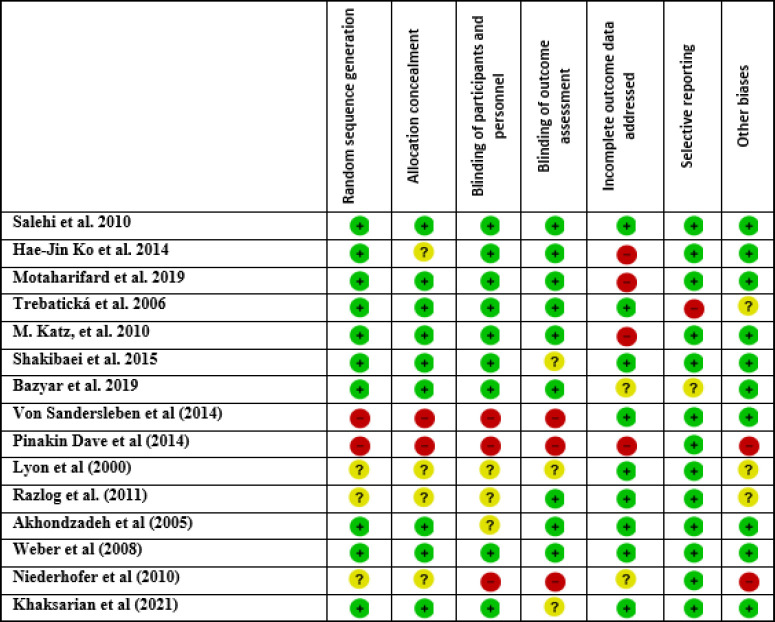
Risk of bias assessment: using the Cochrane risk of bias tool

**Figure 3:**
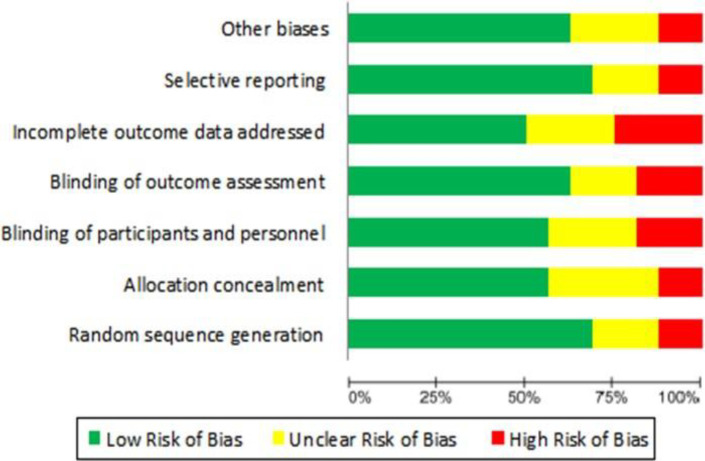
Risk of bias is presented as percentage across all the included studies

The search in all scientific databases resulted in finding 8311 related studies in the field of herbal medicines and ADHD, 3233 of which were duplicates, and 5062 of which were excluded for reasons such as irrelevance, lack of required data, and not found full text. Finally, fifteen eligible articles were included in this study (Figure 1). The characteristics of these included studies are shown in Table 1. Among them, eleven studies were placebo‐controlled RCTs in ADHD patients (with placebo or any pharmaceutical medication). Six of them were conducted in Iran, three in Slovakia, and one in other countries, including the United States, Canada, South Africa, India, Germany, South Korea, Italy, and Israel. A total of 840 people participated in these studies. All included studies reported less female than male participants. The most commonly used diagnostic criteria were the DSM-IV. According to the risk of bias analysis, two studies had not used the random sequence generation approach and the status of randomization in two studies was unclear. The allocation concealment criteria in two studies were not met and these criteria in five studies were unclear. The most common problem among the included studies was incomplete outcome data addressed (four studies). Also, the selective reporting bias observed in the results of one study. More information is shown in Figure 2. Overall, the risk of bias among the included studies was low. The random sequence generation was done in more than 70% of them. Also, allocation concealment and blinding, were considered in more than 50% of the studies (Figure 3).

**Table 1:** Characteristics and results of the included studies

**Author/Year/Country**		**Design/Duration**		**Baseline characteristic**		**Intervention**		**Outcomes**		**Result**	**Conclusion**		
Khaksarian et al. 2021, Iran		RCT/8 weeks		**Treatment group** N: 35, M/F: not mentioned, mean age (years): 10.57±2.56 **Control group** N: 35, M/F: not mentioned, mean age (years): 11.30±2.31.		**Treatment group** *Crocus sativus *L*. *(saffron) capsules at a dose of 20 - 30 mg/d according to the BMI+ MPH **Control group** MPH 0.3 - 1 mg/d at adjusted doses		Parents and teachers completed ADHD-RS-IV at baseline and weeks four and eight.		In both groups, the patients had less symptoms after eight weeks of treatment MPH+saffron had excellent effect on the reduction of symptoms. After four weeks, the average score in the MPH+saffron group was lower than the average total score in the MPH group after eight weeks.	Combination therapy with MPH and saffron is effective in ADHD.		
				Seventy children aged between 6 and 16 years completed the trial									
Baziar et al. 2019, Iran		RCT/6 weeks		**Treatment group** N: 25, M/F: 19/6, mean age (years): 8.2±1.59 **Control group** N: 25, M/F: 21/4, mean age (years): 9.0±2.23		**Treatment group **Saffron capsule 20–30 mg/day based on weight. **Control group** 0.3–1 mg/kg/day MPH at adjusted doses		ADHD symptoms evaluated by teacher and parent ADHD-RS-IV at baseline and weeks 3 and 6.		Changes in symptoms were not significantly different between the two groups. The frequency of adverse effects was similar between two groups.	Treatment with *Crocus sativus *L. displayed the same efficacy compared with MPH.		
				Fifty children aged between 6 and 17 years completed the trial.									
Motaharifard et al. 2019, Iran		RCT/8 weeks		**Treatment group** N: 25, M/F: 16/9 mean age (years): 6.6±1 **Control group** N: 25, M/F: 17/8 mean age (years): 7.5±1.5 Fifty patients aged between 6 and 14 years completed the trial.		**Treatment group** Sweet almond syrup 5 cc/day TID and a therapeutically ineffective tablet as a placebo **Control group **MPH at a dose of 1 mg/kg/day and a therapeutically ineffective syrup 5 cc/day as a placebo		ADHD symptoms were evaluated by parent and teacher ADHD-RS- IV every two weeks for 8 weeks.		There was no significant difference between the two groups regarding the parent and teacher rating scale scores. Both groups showed a decreasing trend in ADHD symptoms.	Sweet almond can be used as an alternative treatment for ADHD		
Shakibaei et al. 2015, Iran		RCT/6 weeks		**Treatment group** N:31, M/F:19/12, mean age (years): 7.8±1.2 **Control group** N:29, M/F:20/9, mean age (years): 8.4±1.4		**Treatment group** *G. biloba *tablet 80-120 mg/day and MPH 20–30 mg/day based on weight **Control group** MPH 20-30 mg/day based on weight and placebo tablets (starch and lactose) in same dosage		ADHD symptoms assessed by parent and teacher ADHD-RS-IV at baseline, 2^nd^ and 6th week of the study.		ADHD-RS-IV parent rating inattention score, the total score and the teacher rating inattention score were reduced among the participants of the treatment group compared to placebo group. The higher response rate was reported on parent rating score in the treatment group than the placebo group.	The *G. biloba *is an effective complementary treatment for patients with ADHD.		
					Sixty patients aged between 6 and 12 years completed the trial								
Sandersleben et al. 2014, Germany		Open clinical pilot study/3 to 5 weeks			N:20, M/F:15/5, mean age (years): 8.2±1.6 Twenty patients aged between 6 and 12 years completed the trial	*G. biloba *special extract up to 240 mg/day based on persistence of symptoms (The initial dose is 40 mg twice daily and increased to 60 and 120 mg twice daily).	Changes in hyperactivity, impulsiveness and aggressive behaviors were measured via the DISYPS-KJ FBB-HKS and FBB- SSV.			Quality of life, ADHD symptoms and CPT performance were improved.	*G. biloba *might be considered as an alternative medicine for ADHD.		
Ko et al. 2014, Korea		RCT/8 weeks			**Treatment group** N:33, M/F:21/12, mean age (years): 10.9±2.2 **Control group **Number:37, M/F:23/14, mean age (years): 10.8±2.4 Seventy children aged between 6 and 15 years completed the trial	**Treatment group** One pouch (40 mL) of KRG extract twice a day. **Control group** One pouch (40 mL) of an identically flavored and identically packaged placebo twice a day	The symptoms of ADHD were evaluated at baseline and week 8 based on DSM-IV criteria. An electroencephalography theta/beta ratio was performed at baseline and week 8. The salivary cortisol and DHEA levels were measured at baseline and weeks 4 and 8.			Inattention/hyperactivity scores among the treatment group was notably reduced at week 8. Theta/beta ratio among the treatment group significantly decreased.	KRG extract may be an effective and safe alternative treatment for children with inattention and hyperactivity/impulsivity symptoms.		
Dave et al. 2014, India		Open-label clinical trial/6 months			N: 27 M/F: 24/3 Twenty-seven children aged between 6 and 12 years completed the trial	One capsule/day, which contained 225 mg of *B monnieri *extract and 125 mg of microcrystalline cellulose	Parent Rating Scale was used to evaluate the ADHD symptom scores at baseline and the end of the study.			*B monnieri *significantly reduced the subtests scores of ADHD symptoms, except for social problems. The symptom scores for restlessness were reduced in 93% of participants, whereas improvement in self-control was observed in 89% of the patients. Symptom scores were reduced for psychiatric problems, impulsivity, learning problems.	*B monnieri *was effective in relieving the symptoms of ADHD.		
Razlog et al. 2011, South Africa		RCT (pilot study)/3 weeks			**Treatment group** Group 1: N: 10 Group 2: N: 10 **Control group** N: 10 The baseline characteristic is not described separately for each group Twenty-seven participants (18 boys and 9 girls) aged between 5 and 11 years, mean age (years):7.93 completed the study	**Treatment group** Group 1: *Valeriana officinalis *(mother tincture) 10 drops/3 times per day after meals for two weeks Group 2: *Valeriana* *officinalis *(homeopathic 3× potency) 10 drops/3 times per day after meals for two weeks **Control group** Placebo, with the same taste and smell, a dose of 10 drops, three times a day after meals for two weeks	Evaluating ADHD symptoms by using Barkley and DuPaul teacher rating scale, the children’s checking task (CCT), and Conner’s parent symptom questionnaires (PSQ), at baseline, first and second week of treatment period and one week after treatment period.			In the MT and 3X groups, an improvement was seen in the participants’ behavior, including attention, anxiety and impulsivity and/or hyperactivity.	The findings of this study showed the positive role of *Valeriana officinalis *in the management of ADHD.		
Salehi et al. 2010, Iran	RCT/6 weeks		**Treatment group** N: 25, M/F: 19/6, mean age (years): 9.1±1.6 **Control group** N: 25, M/F: 20/5, mean age (years): 9.6±2.2 Forty-six patients aged between 6 and 14 years completed the trial			**Treatment group** *G. biloba *capsule 80–120 mg/day depending on weight **Control group **MPH 20–30 mg/day depending on weight		The symptoms of ADHD measured by parent and teacher ADHD-RS- IV at days 0, 21, and 42 of the study.	Signiﬁcant better scores were seen in control group compared to the treatment group. Decreased appetite, headache and insomnia were detected more in the MPH group.		Administration of *G. biloba *for pediatrics ADHD was less effective than MPH.	
Katz, et al. 2010, Israel	RCT/4 months		**Treatment group** N: 73, M/F: 56/17, mean age (years): 9.8±1.5 **Control group** N: 19, M/F: 15/4, mean age (years): 9.3±1.9 Ninety-two patients aged between 6 and 14 years completed the trial.			**Treatment group** 3 ml of CHP include *Melissa ofﬁcinalis*, *Paeoniae alba*, *Withania somnifera, Centella asiatica, Spirulina platensis& Bacopa monieri*, diluted in 50–60 ml of water, 3 times a day **Control group** A dose of 3 ml placebo in 50–60 ml of water was taken 3 times a day		ADHD symptoms measured by TOVA at baseline and post- treatment	Increase of TOVA scores in the treatment group. No changes over time have been seen among the control group. The difference between two groups were statistically signiﬁcant.		CHP improved cognition, attention, and impulse control in the treatment group, which may be a promising alternative treatment for ADHD.	
Niederhofer, 2010, Italy	Open trial/4 weeks		Three male patients aged 14 to 16 years			*H. perforatum *(30 mg per day) and then placebo for 4 weeks.		Conner Scale and the Continuous Performance Test	Improvement ADHD scores for Conners’ inattention, hyperactivity, and immaturity factors.		*H. perforatum *may be a marginally effectual treatment for ADHD	
Weber et al. 2008, USA	RCT/8 weeks		**Treatment group** N: 27, M/F: 20/7, mean age (years): 9.9 **Control group** Number: 27, gender (M/F): 14/13, mean age (years): 9.7 Fifty-four patients aged between 6 and 17 years completed the trial.			**Treatment group** 300 mg of *H. perforatum* three times daily **Control group** Placebo (contained a mixture of rice protein powder and a small amount of activated charcoal)		ADHD RS-IV and CGI scales for improvement and severity were used during study visits at baseline and weeks 1, 2, 4, 6, and 8.	No significant difference was found regarding the change in ADHD symptoms between the treatment and placebo groups. No significant difference was found between the two groups regarding the adverse effects		*H. perforatum *did not improve symptoms of ADHD.	
Trebatická et al. 2006, Slovakia		RCT/4 weeks			**Treatment group** N:44, M/F:37/7, age range (years): (6–14), mean age (years): 9.5 **Control group** N:17, M/F:13/4, age range (years): (6–12) mean age (years) 8.8 Fifty-seven patients completed the study	**Treatment group ** *Pycnogenol *tablet 1 mg/kg/day **Control group** Placebo contained lactose (58 mg) and cellulose (65 mg) in tablet		ADHD symptoms measured by parents with CAP, CTRS, CPRS and Prague Wechsler Intelligence Scale for children (PDW) as well as Modified Wechsler Intelligence Scale for children (WISC). Patients were investigated at the beginning of the trial, after 1 month of treatment and 1 month after termination of treatment.	Following 1-month of treatment with Pycnogenol, scores for hyperactivity and inattention dropped significantly compared to placebo group CTRS scoring after 1 month of treatment with Pycnogenol showed a marginally significant reduction compared to placebo. CPRS scoring at the end of treatment showed marginally significant reduction only for hyperactivity compared to placebo. Relapse of symptoms was seen One month after stop of treatment with Pycnogenol.		*Pycnogenol *could relieve ADHD symptoms in children
Akhondzadeh et al. 2005, Iran		RCT/8 weeks			**Treatment group** N: 17, M/F: 11/6, mean age (years): 9.58±2.09 **Control group** N: 17, M/F: 12/5, mean age (years): 9.05±2.53 Thirty-one patients completed the trial	**Treatment group** *Passiflora incarnata *tablets 0.04 mg/kg/day (twice daily) **Control group** MPH 1 mg/kg/day (twice daily)		Parent and Teacher ADHD Rating Scale used to measure the ADHD symptom scores at 0, 14, 28, 42, and 56^th^ day of the study.	Both groups demonstrated significant clinical benefit after treatment. Decreased appetite and anxiety/nervousness were more common among the control group.		*Passiflora incarnata *may be considered as a treatment for ADHD with tolerable side effect profile.
Lyon et al. 2000, Canada		Open clinical pilot study/4 weeks			N:36, M/F: not mentioned, age range: 3 to 17 years, mean age 10.2±3.7 Thirty-six patients completed the trial	A capsule containing *Panax quinquefolium *extract (200 mg) and *G. biloba *extract (50 mg) twice a day. Twenty-five subjects were taking other medications concomitantly (Ritalin, n = 9; Dexedrine, n = 4, Efamol, n = 2).		ADHD symptoms were measured via CPRS at baseline, 2^nd^ week and 4^th^ week of intervention.	At the end of the study, the Conners' ADHD index and the DSM-IV scales were improved from 44% to74%.		Treatment with *G. biloba *and ginseng may improve symptoms of ADHD.

Outcomes

Ginkgo biloba *L.*

*Ginkgo biloba *L. is an ancient medicinal species native to China, which is mostly known as ginkgo and maidenhair tree. Due to its antioxidant, neurotransmitter/receptor modulatory, and inhibition of platelet activation, this plant has the potential to improve conditions such as impairments in memory and cognitive speed, Alzheimer's dementia, stroke, and aging (Diamond et al., 2000). Lyon et al. (2001) conducted an open-label clinical study to determine the efficacy of a herbal product containing *G. biloba *extract (200 mg *Panax quinquefolium *L. extract and 50 mg *G. biloba *extract) in the treatment of ADHD. They reported that this herbal product could relieve the symptoms of ADHD. However, due to the lack of a comparison group in this study, the results are inconclusive (Lyon et al., 2001).

Uebel-von Sandersleben et al. (2014) conducted an open-label clinical study to evaluate the effect of *G. biloba *special extract on ADHD symptoms in 20 children. The initial dose was 40 mg twice daily and based on persistence of symptoms increased to 60 and 120 mg twice daily. After 3–5 weeks of treatment, an amelioration in ADHD symptoms as well as Continuous Performance Test (CPT) performance and quality of life were reported in children given *G. biloba *(240 mg/daily). No serious adverse events described in this study (Uebel-von Sandersleben et al., 2014).

Shakibaei et al. (2015) compared the effect of a herbal preparation of *G. biloba *and placebo on 60 children and adolescents with ADHD who were receiving methylphenidate (MPH). They assessed ADHD symptoms by parents and teachers using the ADHD Rating Scale IV (ADHD RS-IV). After six weeks of treatment, more reduction was observed regarding parent rating inattention score and total score as well as teacher rating inattention score in the *G. biloba *group. The response rate was also significantly higher with *G. biloba *based on parent rating compared with placebo (Shakibaei et al., 2015).

The study by Salehi et al. (2010) evaluated the effect of *G. biloba *compared to a standard MPH therapy over 6 weeks on 50 ADHD children. At the end of the study, it was found that *G. biloba *in the dosage range of 80–120 mg/day had a lower efficacy compared with MPH. Salehi et al. reported significant changes over time in the assessed ADHD symptoms for the MPH group. Whereas among the *G. biloba *group changes over time were reported to be non- significant (Salehi et al., 2010). Both studies of Salehi et al. (2010) and Shakibaei et al. (2015) reported only mild to moderate side effects (Salehi et al., 2010; Shakibaei et al., 2015).

Pine bark extract

Pycnogenol (French maritime pine bark extract) contains polyphenolic compounds with free radical-scavenging activity against reactive oxygen and nitrogen species, and it is able to create a variety of potential protective effects against chronic and degenerative diseases such as neurodegenerative and neurodevelopmental disorders. Treatment with Pycnogenol reduced hyperactivity of children and catecholamine excretion and improved the antioxidant status of ADHD children (Iravani and Zolfaghari, 2011). There were 3 studies with 165 participants included in this review using Pycnogenol on ADHD.

Trebaticka et al. (2006) evaluated the effects of treatment with Pycnogenol (1 mg/kg/day) for 1 month on 61 participants (aged 6–14 years) compared to a placebo. They reported that Pycnogenol was able to improve ADHD symptoms as well as visual-motor coordination. After discontinuing the use of pycnogenol, a relapse of symptoms was observed in patients. The placebo group showed no amelioration during the study period. The authors did not report any serious side effects. Only an increase in slowness and gastric discomfort in two patients in the intervention group was reported (Trebatická et al., 2006). Due to the lack of determination of primary and secondary results, there is a risk of selective reporting bias in this report. Furthermore, this clinical trial was supported by Horphag Res. Ltd, the manufacturer of Pycnogenol, the risk of other biases is also expected.

In summary, minimal side effects as well as the significant effects of Pycnogenol might confirm that it could be considered an alternative treatment for ADHD, however, more research is needed before a definitive judgment can be made about the overall application of the findings.

 Ginseng

The roots and rhizomes of Korean ginseng (*Panax ginseng *C.A. Meyer) and American ginseng (*Panax quinquefolius *L.) are the main sources of ginseng. It is a herbal medication with diverse pharmacologic activities, including anti- stress effect, improvement on cognitive function, prevention of memory impairment, and antioxidant effect (Baeg and So, 2013). The therapeutic benefits of ginseng in pediatrics ADHD have been studied previously.

Lyon et al. (2001) reported that a herbal product containing *G. biloba *extract (200 mg *Panax quinquefolium *L. extract and 50 mg *G. biloba *extract) could relieve the symptoms of ADHD. This study did not have a comparison group (Lyon et al., 2001).

In another included study, the effects of ginseng compared to a placebo were evaluated on 70 ADHD patients aged 6–15 years old. In the intervention group, 33 patients were given 40 ml of Korean red ginseng (KRG) twice per day. After 8 weeks, a decrease in inattention and hyperactivity scores was recorded in treatment group compared with the control group. Moreover, a decreased electroencephalography theta/beta ratio was observed in comparison with the control group. The two groups did not differ significantly in terms of the frequency of adverse events. There were no serious adverse reactions (Ko et al., 2014).

Prunus dulcis

*Prunus dulcis *(Mill.) D.A.Webb or sweet almond is a nutraceutical and therapeutic agent, which can improve all neurological functions (Sahib, 2014; Karimi et al., 2021). Motaharifard et al. (2019) evaluated the efficacy and safety of sweet almond compared with the standard treatment through a randomized controlled trial on 50 children aged between 6 to14 years. Symptoms were evaluated via ADHD RS-IV. The results of the study showed that sweet almond is as effective as MPH in the treatment of ADHD. Regarding both the parents and teachers ADHD RS- IV, the results showed a significant treatment effect. At the end of the trial, no significant difference was observed between the two groups. The two treatment groups were similar in trend over time and the difference was not statistically significant. However, more studies are needed with larger sample sizes as well as with different doses of the medication and longer follow-up. All observed adverse effects were transient and were not serious. The frequency of reported adverse events was more in patients receiving MPH (Motaharifard et al., 2019).

Melissa officinalis *L*.

*Melissa officinalis *L. or lemon balm is a plant that is native to Iran, southwestern Europe, and Asia (Moradkhani et al., 2010). Katz et al. (2010) tested an herbal compound (its main ingredient was *M. officinalis*) to treat ADHD symptoms in 120 participants. The score of the test of variables of attention was the primary outcome. A high rate of attrition among the placebo group could indicate the high risk of incomplete outcome data in this study. All subtests of the test of variables of attention score for the intervention group between baseline and post-intervention measurement were significantly improved. No significant changes over time were observed in the placebo group. No serious adverse events occurred during the study. Minor side effects happened within both arms of the study (Katz et al., 2010).

Crocus sativus *L*.

*Crocus sativus *L., also recognized as saffron, has traditionally been used for its antispasmodic, anticancer, anticancer, and anticonvulsant properties (Srivastava et al., 2010). The efficacy of saffron as an ADHD treatment has been studied in two studies. Baziar et al. (2019) in a pilot study reported that treatment with saffron showed the same efﬁcacy as MPH (Baziar et al., 2019). Fifty-four ADHD children were randomly

allocated to either MPH or saffron groups. Symptoms were assessed by both teacher and parents via the ADHD-RS-IV at baseline and then the third and sixth weeks of the study. This study showed that changes in ADHD-RS-IV scores during the study period were not signiﬁcantly different between the two groups (Baziar et al., 2019). Another study aimed to evaluate the effectiveness of saffron in the treatment of children suffering from ADHD who were receiving MPH. This showed that after four weeks, the average score assigned by the parents and teachers in the MPH with saffron group was lower than the average total score in the MPH group (p<0.05) (Khaksarian et al., 2021).

Bacopa monnieri

*B. monnieri *-also known as *Herpestis monnieri*, water hyssop, or Brahmi- has been used in the traditional medicine as a tonic for learning, memory, and concentration. This plant has antioxidant activity and protective effects against dopaminergic degeneration. Treatment with 225 mg Bacopa extract per day for 24 weeks showed a significant alleviation of the symptoms of ADHD in children. In this study, the score for restlessness was improved among more than 90% of participants. Also, the score for self-control as well as attention-deficit symptoms was improved in 89% and 85% of participants, respectively. Furthermore, impulsivity, learning and psychiatric problems were improved (Dave et al., 2014).

Valeriana officinalis *L*.

*Valeriana officinalis *L. or valerian is a medicinal herb with known sedative and antispasmodic effects. The efficacy of this plant in the treatment of ADHD has been evaluated in a pilot study (Razlog et al., 2011). The study included 30 children aged 5 to 11 years who were given valerian for two weeks and three times a day. The authors reported an improvement in ADHD symptoms which were measured via Children's checking task (CCT), Conner's parent symptom questionnaire (PSQ), and the Barkley and DuPaul teacher rating scale. Because the authors did not mention randomization or blinding in the article, there is a possibility of bias in the results of this study, so further studies are needed to confirm the results of such a study and to decide on the effectiveness of valerian in treatment. No serious adverse events or side effects were reported by the authors.

Passiflora incarnata *L.*

Recent preclinical studies have displayed potential effects of *Passiflora incarnata *L. or passion flower on anxiety, insomnia and ADHD (Miroddi et al., 2013). The effect of passion flower on ADHD symptoms has been investigated in 34 children with ADHD; the treatment group received passion flower twice a day. The control group received MPH twice a day. No significant difference was reported based on parent and teacher rating scores. Moreover, the side effects were less common among the passion flower group compared with placebo group. Although anxiety and loss of appetite were recorded in the control group more than the intervention group, the authors reported no serious side effects (Akhondzadeh et al., 2005). The blinding of participants and assessors remained unclear in this study.

Hypericum perforatum *L.*

*Hypericum perforatum *L., also known as St John's wort, is a plant in the Hypericaceae family that has an influence on certain CNS neurotransmitters (Barnes, et al., 2001). A preliminary study by Niederhofer (2010) reported an improvement in ADHD symptoms in 14-16 years old patients who were administered St. John’s wort (Niederhofer, 2010). However, a randomized trial by Weber et al. (2008) aimed to study the safety and efﬁcacy of a *H. perforatum *in the treatment of ADHD found that 8 weeks of *H. perforatum *treatment (300 mg/day) did not reduce ADHD symptoms in 54 ADHD children (Weber et al., 2008). The ADHD RS-IV was used to evaluate symptoms rated by parents. The severity of symptoms was also measured via the Clinical Global Impression Improvement Scale (CGI). The frequency of adverse events as well as side effects was equal between the two groups.

Based on these findings, further studies are needed to assess the efficacy of *H. perforatum *in the treatment of ADHD. Although the above studies did not report adverse effects of *H. perforatum*, investigation on the safety of this herbal remedy is still required.

In summary, the results of included reports showed that most of the mentioned herbal remedies had positive effects in alleviating the symptoms among the children and adolescents with ADHD. Another point was about the side effects of the studied herbal medicines. According to the results, they had minimal side effects, which was one of the advantages of using them in combination with current treatment options. In addition to the beneficial therapeutic effects, having ignorable side effects makes them more welcome and increases their tolerability, which can be seen in the case of herbal medicines and phytochemicals.

## Discussion

The findings of the current systematic review show positive but inconclusive evidence of herbal remedies in the treatment of ADHD. Seven of the fifteen included studies had a high-risk bias domain. Thus, despite the positive findings, it is difficult to conclude that herbal medicines and phytochemicals are effective treatments for ADHD in children and adolescents, and the seemingly promising results should be interpreted with caution. The findings of this study are relatively consistent with previous systematic reviews of herbal and nutritional products in the treatment of ADHD (Sarris et al., 2011; Anheyer et al., 2017). Except for St. John’s wort which does not show positive results, almost all other herbal medicines revealed mainly positive evidence and may provide a beneﬁcial effect on reducing ADHD symptoms.

The results for *G. biloba *and Pycnogenol were based on multiple studies but not conclusive. While Pycnogenol and *G. biloba *are promising herbal medicines in the management of ADHD symptoms, more investigation with a longer duration is essential to prove the worth of these medicines as an option in the treatment of ADHD. However, in each case, based on only one study, the result for *M. officinalis *and ginseng were positive but, it is difficult to make any decision about the efficacy, and more studies are required to assess the efficacy of these medicines in treating ADHD. Further studies also are required to confirm the safety and efficacy of Bacopa as an ADHD treatment.

The potential effect of ginseng can be considered an alternative to ADHD treatment, due to its low side effects and high efficacy. The neuroprotective as well as the antioxidant effects of ginseng, which could increase the dopamine and norepinephrine levels have been shown (Ong et al., 2015). The efficacy of treatment by Passion flower, saffron, and sweet almond in pediatrics ADHD was similar to that reported for MPH. The safety and lower side effects of these herbal medicines compared to MPH could be considered the main advantage. Passion flower has neuroprotective effects and could alter the serotonergic neurotransmission in the brain (Jawna‐Zboińska et al., 2016). Boron is a trace element found in sweet almonds; some studies have shown that boron plays a key role in improving cognitive performance (Penland, 1994; Pizzorno, 2015). Saffron affects glutamatergic and monoaminergic systems (Sarris, 2007; Curatolo et al., 2009) and is generally a safe medication (Schmidt et al., 2007).

All the studies reported no serious adverse events or severe side effects. Seven of the fifteen studies assessed in this review, including Akhondzadeh et al. (2005), Trebatická, et al. (2006), Weber et al. (2008), Katz et al. (2010), Salehi et al. (2010), Razlog et al. (2011), and Shakibaei et al. (2015) were also included in the review of Anheyer et al. (2017) were consistent with our findings regarding the *G biloba, St. John's wort, M. ofﬁcinalis*, *P. incarnata*, and the *V. ofﬁcinalis *(Anheyer et al., 2017). In the present study, the evidence for saffron, ginseng, *Bacopa monnieri*, and sweet almond was furthermore examined.

The limitation of this systematic review was about the rarity of eligible clinical trials. For some herbal medicines, only one eligible study could be identified, and despite a comprehensive search, we eventually included only a small number of randomized, controlled clinical trials. Also, further randomized, controlled clinical trials are required to confirm the effectiveness of herbal treatment on ADHD in children and adolescents. In addition, it seems that researchers in this field need to pay special attention to the Consolidated Standards of Reporting Trials (CONSORT) in presenting the results of primary studies. Despite the acceptable methodological quality of most studies, the small sample size needs to be taken into consideration as a limitation. Moreover, the unclear randomization and allocation concealment methods, incomplete reporting of outcome data in some studies were among other limitations.

A higher frequency of male participants was found in all of the included studies. In addition, it should be noted that the countries in which different studies have been conducted are culturally different and largely heterogeneous. Finally, we have included only the published studies in English language in the review.

In summary, the studies included in the current systematic review show that herbal medicines appear to be relatively safe and with limited side effects and providing potential efficacy in amelioration of ADHD. Because there are not yet enough RCTs of herbal remedies on ADHD, no specific recommendations would be possible. We hope that the results of the 

present study, in addition to assisting physicians in evidence-based decision- making, will be useful both in providing clinical evidence-based guidelines and in encouraging researchers to conduct new, high-quality randomized trials in this field.

## Conflicts of interest

The authors have declared that there is no conflict of interest.
